# The Prevalence of Sensitive Skin

**DOI:** 10.3389/fmed.2019.00098

**Published:** 2019-05-17

**Authors:** Miranda A. Farage

**Affiliations:** The Procter & Gamble Company, Mason Business Center, Mason, OH, United States

**Keywords:** self-perceived sensitive skin, gender differences, age differences, facial skin, anatomic sites, cultural factors, barrier dysfunction, neurosensory dysfunction

## Abstract

Sensitive skin has been described as unpleasant sensory responses to stimuli that should not provoke such sensations. Objectively measurable signs of irritation are not always present in individuals with sensitive skin, however, subjective sensory effects such as, itching, burning, stinging, tightness, and dryness, are consistently present. Given the subjective nature of the phenomenon known as sensitive skin, surveys have been a popular approach to evaluating the prevalence of this condition among the general population, and a number of them have been conducted worldwide. Overall, ~60–70% of women and 50–60% of men report having some degree of sensitive skin. However, there are differences between populations in various geographies, and perceptions of sensitive skin at specific anatomic sites. This article is a review of survey data on the prevalence of self-declared sensitive skin in various geographies, among different gender and age groups, and at various anatomic sites. In addition, we review the factors that may contribute to sensitive skin, and the physiological characteristics associated with this condition, including impaired barrier function and heightened neural reactions.

## Introduction

The development of consumer beauty, health and household products routinely includes intensive premarket product testing intended to ensure that any marketed product is free of irritant potential. Nonetheless, it is not uncommon for post-marketing surveillance personnel to receive reports of unpleasant sensory reactions to such products not predicted by even the most robust development methodology (1). The sensory reactions of consumers strongly influence purchasing decisions. In fact, of consumers who claimed unusually sensitive skin, 78% avoided specific products due to prior experiences of unpleasant sensory effects with their use (2). These adverse sensations are usually transient and unaccompanied by classical visible signs of irritation (3). The underlying mechanism is neither immunological nor allergic (4).

Recently, a special interest group on sensitive skin in the International Forum for the Study of Itch (IFSI) has defined sensitive skin as follows (5).

“*A syndrome defined by the occurrence of unpleasant sensations (stinging, burning, pain, pruritus, and tingling sensations) in response to stimuli that normally should not provoke such sensations. These unpleasant sensations cannot be explained by lesions attributable to any skin disease. The skin can appear normal or be accompanied by erythema. Sensitive skin can affect all body locations, especially the face.”*

This definition of sensitive skin was decades in development. The concept of sensitive skin arose in the 1970s with the observation that despite the fact that previous safety evaluations had found no evidence of toxicity, some patients reported stinging sensations upon using a particular sunscreen that contained a derivative of P-aminobenzoic acid (6). A variety of methods have been used to identify individuals with sensitive skin (Table 1). These have included tests to identify individuals with specific sensory reactions, such as stinging or burning, and traditional tests for irritation with enhanced means of detecting inflammatory responses. Each of these tests has significant limitations. For example, an individual with sensitive skin may respond to one chemical that induces stinging, such as lactic acid, but be non-responsive to other sting-inducing materials. Enhanced detection methods may be able to identify otherwise subclinical irritation reactions, but those reactions may not be associated with sensory responses. Further, such enhanced detection methods tend to require specialized equipment and/or training.

**Table 1:** Some examples of methodologies used to identify sensitive skin.

**Methodology**	**Sensory affect evaluated**	**Physical effect evaluated**	**Relevant irritants**	**Advantages**	**Disadvantages**	**References**
Lactic acid	Stinging	None	Cosmetics, other personal preparations meant to be left on	Sensitive and specific (may be positive in 90% of women who claim sensitive skin)	Does not predict sensitivity to other irritants	(7)
Capsaicin	Stinging	None	Cosmetics, other personal preparations meant to be left on	Sensitive, detection threshold well correlated (inversely) to perception of sensitive skin	Does not predict sensitivity to other irritants	(8)
Sodium Lauryl Sulfate	Burning	Erythema	Industrial exposures, cleaning products	Cheap, quick, reliable assessment of individual susceptibility to specific irritant	Sensitivity to one irritant not predictive of general sensitivity, relationship to sensitive skin in question	(9)
Cross-polarized light	None	Subclinical erythema	Any potential irritant	Permits detection of physical changes not apparent by standard visual scoring, noninvasive	Requires specialized equipment	(10)
Infrared thermographic scanner	None	Temperature increases resulting from inflammatory processes related to skin injury	Any potential irritant	Non-invasive, objective, quantitative	Requires specialized equipment	(11)
Sebutape®	None	Measurement of cytokines produced by injured skin	Any potential irritant	Noninvasive, objective, quantitative, potentially very sensitive	Requires training, specialized equipment. Utility for sensitive skin still unassisted	(12)

Further complicating the use of irritant testing in identifying sensitive skin is the observation that there is profound interpersonal variability in individual responses to specific irritants (13, 14), even among chemicals with similar modes of action (15). Sizeable variation exists within the same individual at different anatomic sites (14), and even at the same anatomical site on symmetric limbs (9). In addition, many people who profess sensitive skin do not predictably experience irritant reactions, whereas some who describe themselves as non-sensitive react strongly to tests of objective irritation (16).

Traditional irritant-testing methodologies have not proven to be good predictors of adverse sensory reactions. As a result, the investigation of the phenomenon of sensitive skin has progressed using epidemiological investigations implementing surveys conducted of the general population. Such investigations rely on the responders' self-perceived adverse reactions to various products, chemicals, and other potential stimuli. When evaluating such results, it is important to consider that self-reported data can have significant drawbacks. Some patients may experience adverse sensations that are related to underlying dermatologic conditions such as rosacea or seborrheic dermatitis. These conditions can also produce stinging sensations that can be interpreted as sensitive skin (7, 17). There are also psychological disorders characterized by similar symptoms, such as cosmetic intolerance syndrome (18). Further, subject responses can be significantly impacted by the specific wording of survey questions. For example, many questionnaires used in some of the surveys ask a yes/no question with regard to sensitive skin, with no emphasis on particular anatomic sites. Other survey instruments are more specific, drawing the responders' attention to a specific body site, such as the face, genital area, or scalp, and asking a responder to rate the degree of sensitivity (e.g., as non-sensitive, slightly, moderately, or very sensitive).

## Prevalence of Sensitive Skin

### Self-Declared Sensitive Skin in Various Geographies

Sensitive skin was initially believed to be an unusual reaction evidenced in only a small subset of individuals. However, over the last two decades, surveys on sensitive skin have been conducted in over 20 different countries on 5 continents. This research has demonstrated that individuals with sensitive skin represent over half the population.

Table 2 provides an overview of published surveys in which the responders were asked about generalized sensitive skin. For those studies in which the responders were asked to rate the degree of sensitive skin (e.g., “very,” “moderately,” “slightly,” or “not” sensitive), the overall average number of individuals in the general population who claimed some degree of sensitivity (e.g., “very,” “moderately,” or “slightly” sensitive) was about 70%. Individual study results varied from a low prevalence of about 23% to over 90%. If we consider only those individuals who rate their skin sensitivity “moderate” or “very,” the average was about 46%, with a range of prevalence from 7% to over 70%.

**Table 2:** Prevalence of self-declared sensitive skin in various geographies.

**Country**	**Year^**a**^**	**Number of subjects^**b**^**	**Subjects claiming sensitive skin**			**References**
			**Classification question (%)^**c**^**		**Yes/No question^**d**^**	
			**Any degree of sensitivity (%)c1**	**Top two classifications (%)c2**	**Yes responders (%)**	
**North America**						
USA	2007	994 total	na	44.6	na	(19)
		499 women	na	50.9	na	
		495 men	na	38.2	na	
USA	2006	29 incontinent women	82.8	58.6	na	(20)
		42 age matched control	76.2	26.2	na	
USA	2009	1,039 total	68.4	27.9	na	(21–23)
		869 women	69	28.9	na	
		163 men	64.4	23.3	na	
USA (Mississippi)	2013	89 women	77.5	42.7	na	(24)
**South America**						
Brazil	2014	1,022 total				(25)
		women	80.4	45.89	na	
		men	53.06	22.49	na	
Mexico	2011–2012	246 total	na	na	36	(26)
		168 women	na	na	42.2	
		78 men	na	na	23	
**Europe**						
England	2001	2,316 total	na	na	49.95	(27)
		2,058 women	na	na	51.4	
		258 men	na	na	38.2	
France	March, 2004	1,006 total	80.8	52.1	na	(28)
		women	85.5	59.3	na	
		men	74.8	43.7	na	
France	July, 2004	1,001 total	86.7	59.3	na	(29)
		women	91.2	69.3	na	
		men	77.9	45.8	na	
France	2017	5,000 total	na	59	na	(30)
		2,557 women	na	66	na	
		2,443 men	na	51.9	na	
Germany	1999	420 total	75.2	62.3	na	(31)
		258 women	82.6	53.9	na	
		162 men	63.5	36.4	na	
Greece	2005	25 atopic women	100	80	na	(32)
		25 women with non-related complaints	64	16	na	
Italy	2004	2,101 total (88.5% were women)	na	na	59.9	(33)
Netherlands	2013-2014	431 total	na	na	40.8	(34)
		258 women	na	na	75.9	
		184 men	na	na	24.1	
Netherlands	2014	278 women	na	na	45.7	(35)
		121 premenopausal	na	na	41.9	
		55 perimenopausal	na	na	51	
		102 post-menopausal	na	na	47.3	
Europe (total)	2009	4,506 total	74.7	38.1	na	(25, 36)
Belgium	2009	500 total	60	26	na	
France	2009	1,006 total	82	52	na	
Germany	2009	500 total	59	35.8	na	
Greece	2009	500 total	70	31	na	
Italy	2009	500 total	90.6	54.6	na	
Portugal	2009	500 total	86	29.6	na	
Spain	2009	500 total	88	33	na	
Switzerland	2009	500 total	59	31	na	
**Central and Eastern Europe**						
Russia	2014	1,500 total				(25)
		women	85.84	50.06	na	
		men	66.9	25.39	na	
**Asia**						
China	2009	408 women	23	7	na	(37)
China (urban dwellers)	2009	9,154 total	39.53	12.79	na	(38)
		5,223 women	na	15.93	na	
		3,931 men	na	8.62	na	
Japan	2011	1,500 total	na	54.5	na	(39)
		777 women	95.6	56	na	
		723 men	93.5	52.8	na	

*na, not available*.

a *If year of study is not given, the date corresponds to the year of publication*.

b *Subjects taken from the general population unless otherwise indicated*.

c *Most studies asked responders to rate the severity of sensitive skin, e.g., very, moderate, slight or none. c1, average of all severity (very, moderate and slight); c2, average of very and moderate*.

d *Some studies asked responders to agree or disagree with the statement, e.g., I have sensitive skin*.

Berardesca et al. stated that ~70% of the population consider themselves to possess the characteristics of sensitive skin, with about 50% of these patients demonstrating uncomfortable symptoms (40). In a 2016 publication, Misery et al. cited a prevalence rate of about 40% worldwide, comparing responders having “sensitive” or “very sensitive” skin to those having “not very sensitive” or “not sensitive at all” skin (3). These estimations are close to the average prevalence from published surveys shown in Table 2, where about 66% have some degree of sensitivity and about 37% judged their skin to be “moderate” or “very” sensitive.

An exception to the high prevalence rate of sensitive skin presented in Table 2 is China. In a 2009 survey of 408 women from the general population, we found that only 23% of women reported some degree of sensitive skin, and only 7% classified their skin as “moderately” or “very” sensitive (37). It is noteworthy that this survey used the same questionnaire as other surveys we conducted in various geographies, therefore, differences in the wording and possible responses to the questions were not a contributing factor to this difference. The clear difference in the perceived skin sensitivity between China and other geographies may be in part due to cultural factors. An epidemiological study conducted in Europe lends support to the importance of a cultural component. A comparison of self-reported skin sensitivity among 500 subjects in each of eight European countries found dramatic differences between national populations that are genetically very similar (36). For example, 80 to 90% of individuals surveyed in Portugal, Italy, and Spain, reported at least some skin sensitivity. In contrast, individuals surveyed in Germany, Belgium, and Switzerland reported 50–60%. The authors proposed this difference might be related to more fashion and beauty-related advertising in specific European countries.

Another study was conducted in China in 2009 among 9154 subjects from large urban areas. Xu et al. found 39.5% of the entire population (both genders) reported some degree of sensitive skin. Among the women in the survey, only 15.9% classified their skin as “moderately” or “very” sensitive (38). These authors emphasized that the majority of their study population were phototype IV (over 86%). This darker skin type would be expected to lower the prevalence of sensitive skin to some degree. Further, the authors pointed out a significant cultural component. “Sensitive skin” is a new term in China and has been used only in recent years. Older Chinese subjects may have been less clear on the meaning of sensitive skin and, therefore, less likely to categorized themselves as such. Supporting this notion was the observation that the total prevalence of very sensitive and sensitive skin gradually decreased with age. In men, about 11% of age 25 years or less classified themselves as having the “very sensitive” or “sensitive” skin, compared to about 7% of men ≥50 years. For women, these percentages were about 19% of age 25 years or less, compared to about 12% of women ≥50 years.

Mexico is another country with a relatively low prevalence of subjects from the general population claiming sensitive skin (36%) (26). A possible explanation for the lower prevalence in this study was proposed by the author. It has long been recognized that the fair skin phototype is more commonly associated with self-reported sensitive skin compared to the darker skin phototype (19, 41). This was confirmed in the study in Mexico where 22 of 37 (over 59%) of subjects with phototypes II and III claimed sensitive skin, compared to 67 of 209 (32%) of subjects with phototypes IV and V. Differences in the self-diagnosis of sensitive skin also may be related to cultural differences, such as a lower interest in using cosmetic products or reporting adverse reactions to them (26).

### Sensitive Skin at Different Anatomic Sites

For many, the term sensitive skin is synonymous with sensitive facial skin. The face is highly innervated; therefore, adverse sensations may be experienced more acutely. Facial skin comes into contact with a wider variety of products compared to some other anatomic sites, such as, cleansers, cosmetics, and shaving products. Since the face is typically uncovered, this site is also exposed to greater extremes in weather, climate changes, and other environmental insults. Further, individuals may be more aware of appearance and visual signs of irritation on the face and, therefore, likely to perceive their facial skin as more sensitive.

Several epidemiologic studies have specifically probed responders about sensitive skin at anatomic sites other than the face (Table 3). Overall, these investigators have found that the prevalence of perceived sensitive skin of the face tends to be higher than other anatomic sites. However, other body sites cannot be ignored with regards to sensitive skin. Interestingly, in one of our studies we evaluated responses to a sensitive skin questionnaire by comparing the severity of perceived skin sensitivity in general (i.e., “very,” “moderate,” “slight” or “none”) to that of perceived sensitivity at specific anatomic sites (i.e., face, body, and genital area) (21). When describing the severity of sensitivity of facial and body skin, we found that most participants gave responses that were either consistent with the perception of sensitive skin in general (60.7 and 68.4% of responders, respectively) or varied by only one degree of severity (36.7 and 31.3% of responders). When describing the skin in the genital area, the pattern was slightly different. Less than half of the subjects gave the identical description of skin sensitivity in general and of the genitalia (46.2%). Subjects tended to describe their genital skin as less sensitive than their skin in general. This demonstrates the importance of identifying specific anatomic sites in survey instruments designed to determine the prevalence of self-perceived sensitive skin.

**Table 3:** Prevalence of self-declared sensitive skin at specific anatomic locations.

			**Sensitive skin**					
			**Any degree (%)^**b**^**					
**Country**	**Year^**a**^**	**Number of subjects**	**General**	**Face**	**Scalp**	**Genital**	**Body**	**References**
USA	2009	1,039 total	68.4	77.3		56.3	60.7	(21)
		869 women	69	78.6		58.1	60.2	(22)
		163 men	64.4	68.1		44.2	62	(23)
USA (Mississippi)	2013	89 women	77.5	79.8		57.3	74.2	(24)
USA	2006	29 incontinent women (≥50 years old)	82.8	86.2		86.2	69	(20)
		42 age matched control	76.2	82.9		68.3	65.9	
China	2009	408 women	23	20		6	9	(37)
England	2001	2,316 total	50^c^	34^c^	23.5^c^			(27)
		2,058 women	51.4	34.6	23.3			
		258 men	38.2	29.1	25.4			
France	2009	2,117 total			32.2			(42)
		women			35.6			
		men			29.1			
France	2004–2005	400 women		85	36			(43)
Netherlands	2014	278 women	45.7	53.6		10.8		(35)
		121 premenopausal	41.9	62		8.3		
		55 perimenopausal	51	54.5		12.7		
		102 post-menopausal	47.3	43.1		12.7		
Korea	2013	1,000 total		89.4				(44)
		507 women		91.71				
		493 men		87.02				
USA	2012	310 men		54.5				d
Germany	2012	301 men		57.1				
Italy	2012	300 men		81.7				
Spain	2012	300 men		72.3				
United Kingdom	2012	302 men		59.3				
Poland	2012	300 men		74.3				
Russia	2012	304 men		74.7				
Turkey	2012	300 men		82.3				
Japan	2012	304 men		50.3				
Korea	2012	304 men		65.8				
Australia	2012	301 men		51.2				
	**Sensitive skin, Any degree (%)**^**b**^							
	**France** **(****43****)**	**Netherlands** **(****35****)**						
**Anatomic site**	**400 women**	**278 women**	**121 premenopausal**	**55 perimenopausal**	**102 post-menopausal**			
Hands	58	27.3	23.1	29.1	31.4			
Feet	34	17.7	11.6	25.5	20.6			
Neck	27	6.9	8.3	7.3	4.9			
Back	21	9.7	9.9	10.9	8.8			
Torso	23							
Chest		16.9	13.2	25.5	16.7			
Legs		27.7	23.1	27.3	33.3			

a *If year of data collection is not given, the date corresponds to the year of publication*.

b *Includes any degree of general sensitivity, for example: very, moderate, or slight*.

c *Percentages not reported, but interpreted from other data in publication*.

d *Unpublished data from a Procter & Gamble marketing survey*.

The face has demonstrated to be the most common site of skin sensitivity (Table 3), predictable physiologically due to the larger and multiple number of products used on the face (particularly in women), a thinner barrier in facial skin, and a greater density of nerve endings (18). The nasolabial fold was reported to be the most sensitive region of the facial area, followed by the malar eminence, chin, forehead, and upper lip (15, 43). Saint-Martory found hand, scalp, feet, neck, torso, and back sensitivity followed facial sensitivity in descending order of prevalence (43). Significant numbers of individuals experience sensitivity of the scalp (42, 45).

In a study of 1,039 men and women, 56.2% reported sensitivity of genital skin (21), an area of particular interest since it is formed partially from embryonic endoderm and therefore differs from skin at other body sites (16). Significantly more African-Americans than Caucasians (66.4%, *p* < 0.0001) claimed sensitivity of this area. Rough fabrics were found to be the most common offender for sensitive skin in the genital area (46).

Earlier, mention was made of the variation in resulting prevalence estimates from the different studies presented in Table 2. Such variation can also be observed in the data summarized in Table 3. There are a number of explanations for the differences in the findings of various studies with regard to the prevalence of sensitive skin. First, no standard survey instrument was used. Rather, each investigator used a survey instrument tailored to his or her specific interests. Therefore, there is a great deal of variability in the wording of questions regarding self-perceived sensitive skin. Another reason why the prevalence of sensitive skin may vary is related to the year in which the study was conducted. There is evidence that reported prevalence of self-perceived skin sensitivity have increased steadily over time (21). Therefore, earlier studies may record a lower prevalence.

Studies where skin sensitivity prevalence has been reported separately for men and women are fairly consistent in showing that a higher percentage of women perceive they have sensitive skin (Table 2). In a study reported in 2010 we found that the perception of skin sensitivity tended to shift toward less severe perceived reactions for the men, with a lower percentage reporting “very sensitive” compared to women, and a higher percentage reporting “slightly sensitive” (22).

The gap between men and women may be closing. As skin care and personal grooming products specifically for men become more common, male consumers will likely focus more on skin condition. In a worldwide survey of dermatologists, most practitioners indicated they agreed or strongly agreed that they have noticed an increase in male patients reporting sensitive facial skin over the past 5 years (47). In an unpublished companion marketing survey conducted among men from 11 different countries, more than 50% of subjects claimed sensitive skin of the face (Table 3).

As people age, certain marked changes occur in the structure and function of skin. Skin becomes thinner and drier, with a loss of subcutaneous fat (48). Characteristic morphological changes occur in the cell types in the epidermis, and physiological changes in aged skin include changes in biochemistry, neurosensory perception, permeability, and vascularization (48). It is reasonable to expect that skin would become more sensitive with age. However, this has not been a consistent finding in surveys on sensitive skin. In a study reported on 2011, we found that perceptions of sensitive skin in general and of the face and body were not dependent on age. The perception of sensitive genital skin was dependent on age for the women, but not for the men (46). Hernández-Blanco et al. reported no substantial difference in self diagnosed sensitive skin among different age groups (26). In a 2017 study conducted among 5,000 subjects in France, Misery et al. concluded that sensitive skin was reported more frequently among individuals under 35 years of age than among individuals 35 and older (30).

A publication appearing in print after this article was drafted reported on the prevalence of perceived sensitive facial skin in India (49). In this study, ~64% of men and 73% of women reported some degree of sensitivity (“very,” “sensitive” or “slightly sensitive”). When only “sensitive” or “very sensitive” skin was considered, the proportion was 27.9% of men and 36.7% of women, which was a significant difference (*p* < 0.001).

## Contributing Factors Associated With Sensitive Skin

### Individual Characteristics and Cultural Factors

A number of characteristics have been associated with an increased likelihood of sensitive skin (Table 4). In most studies, sensitive skin is self-reported more often in women than in men. The thickness of the epidermis was observed to be greater in males than in females, which may provide a biological explanation for greater sensitivity among women (52). However, for the most part, irritant testing finds no differences in reactivity (14). Women tend to use more products, especially on the face, increasing potential exposures to materials that may trigger unpleasant sensations.

**Table 4:** Some factors contributing to sensitive skin.

**Factor**	**References**
Gender	(27, 31)
Menstrual cycle	(35)
Age	(50)
Fair skin, susceptible to sunburn	(27)
Susceptibility to blushing and/or flushing	(27)
Skin pigmentation	(51)
Atopy	(32)
Incontinence	(20)
Environmental and external factors	(23)
Cultural expectations in technologically advanced countries	(31)

Hormonal differences that may produce inflammatory sensitivity in females have also been demonstrated (9, 53). The signs and symptoms associated with sensitive skin have been reported to occur in conjunction with the menstrual cycle as well as subsequent to a cornucopia of possible triggers like weather conditions, air conditioning, cleaning products, personal care products, and clothing (43). In a recent study, Falcone et al. reported that, about half of premenopausal subjects perceived that their menstrual cycle affected their skin (35). Further, among post-menopausal women claiming sensitive skin, over 70% perceived their skin sensitivity increased after menopause.

As mentioned earlier, the physiological changes of aging would also ostensibly predispose individuals to skin irritation (50). A study of sensitive skin in 1,039 subjects in Ohio found those over 50 were more likely to claim sensitive skin than younger adults, particularly in the genital region (50). However, clinical assessment of the erythematous response to irritants in older people suggests a decrease in sensitivity with age (50).

Skin types and ethnicity are known to include pronounced differences in skin structure (54) and susceptibility to specific test irritants (9, 55). A large epidemiological study conducted to determine the incidence of self-reported sensitive skin reported no observed racial differences in prevalence (2). In contrast, a subsequent study the incidence of sensitive skin among African-Americans was higher than that reported by Euro-Americans (*p* = 0.0096) (22).

Some observable specific differences between ethnic groups were reported in epidemiological studies. Euro-Americans, relative to other ethnic groups, were found to have higher susceptibility to wind (2). Asians had higher sensitivity to spicy food, and Hispanics had relatively less reactivity to alcohol (2). African-Americans were more likely to report sensory response to stimuli, while Caucasians more often reported visual effects (23). African-Americans of both genders were more likely to report sensitivity in the genital area than other groups (*p* = 0.0008) (21).

Topical health and beauty products and weather conditions are commonly associated with self-reports of sensitive skin (2, 56). Sensitive skin has also been reported to result from environmental conditions (e.g., sun exposure, hot weather, cold weather, dry air, humidity, wind, air conditioning), health and beauty products (e.g., soap, shampoo, hair color, other hair products, eye cosmetics, facial cosmetics, facial moisturizers, facial astringents, facial cleansers, perfume, fragrances, body moisturizers, anti-aging creams, sunscreen, deodorants, antiperspirants, talc), household items (e.g., cleaning products, dishwashing liquid, laundry detergent, fabric softeners), personal hygiene products (e.g., menstrual pads, pantiliners, incontinence pads, tampons, feminine wipes, douching products, toilet paper), and garments (e.g., underwear, other clothing, rough fabrics). Using a comprehensive questionnaire in China, a population with a comparatively low prevalence of self-perceived sensitive skin, every possible trigger suggested was claimed by at least a few respondents. The perceived prevalence of sensitive skin has been shown to be related to weather both temporally (significantly more women in France reported skin sensitivity in summer than in winter) (28) and geographically (women in China report more sensitivity to hot weather, while women in the USA report more sensitivity to cold) (37).

### Cultural Influences in the Perception of Sensitive Skin

Differences in geography are another cause for variability in reported prevalence rates. In a series of studies conducted in Europe in 2009, Misery et al. found that the occurrence of skin sensitivity was different in various countries even though the European population is very crossbred (25, 36). In France, Italy, Portugal and Spain, ~80–90% of the subjects claimed some degree of skin sensitivity. In Belgium, Germany, Greece and Switzerland the reported prevalence was ~60–70%. Seasonal weather patterns may contribute to these differences.

Lifestyle influences based on culture undoubtedly have an impact on the perception of sensitive skin. Cultural practices produce widely different exposures to potential irritants (8). For example, hygiene practices (use of douches, perfumes, medications, antifungal medications, shaving, and contraceptives) are the most common cause of vulvar irritation (53). Older women were observed in one study to be more likely to report irritation due to incontinence products than younger women, who were more likely to report irritation due to tampons (50)–findings almost certainly based on culturally-driven levels of exposure. Asians in one San Francisco study evidenced a greater skin reactivity to spicy food than Caucasians (2), another finding most likely related to a culturally higher exposure to spicy food.

Interestingly, the description of sensitive skin differs between ethnic groups. Caucasians claim visual (redness/swelling) effects vs. African-Americans and Asians who claim more sensory (burning/stinging) effects (23).

The percentage of people who perceive themselves to have sensitive skin, however, has steadily increased in the US and Europe with the media attention paid to it. This is particularly evident among men, most likely related to an increase in the marketing of sensitive-skin products to men, driving a cultural acceptance of male sensitive skin (22, 47). The fact that the majority of individuals who claim sensitive skin are women in the industrialized world also tends to support at least some psychosocial component.

### Physiological Observations

Sensitive skin appears to include a set of seemingly disparate physiological characteristics observed by a number of investigators over the years. Examples are listed in Table 5. However, these observations have provided clues to the underlying cause of sensitive skin. Potential causal physiological pathways were recently reviewed by Misery et al. (3). Alterations of epidermal barrier function and neurosensory dysfunction are strongly associated with sensitive skin.

**Table 5:** Some physiological characteristics associated with sensitive skin.

**Alterations in barrier function**	**References**
Changes in Transepidermal Water Loss (TEWL) indicating impairment of the cutaneous barrier	(57)
Thin stratum corneum	(54, 55, 58, 59)
Decreased hydration of stratum corneum	(51, 56, 60, 61)
Decreased lipids	(60, 62–66)
Decreased ceramides	(67)
Increase neutral lipids and decreased sphingolipids	(68)
Increased sweat glands	(55)
**Neurosensory function**	
Increased epidermal innervations	(69, 70)
Decrease of intraepidermal nerve fiber density (peptidergic C-fibers)	(4)
Heightened neurosensory input	(8, 60)
Upregulation in expression of TRPV1	(71)
Genetic variation in TRPV1 is associated with susceptibility to capsaicin	(72)

#### Alterations in Barrier Function

Early studies suggested a link between sensitive skin and a disruption in barrier function (9, 62, 73–75). However, attempts to find significant and reproducible differences between sensitive skin subjects and controls via measurements of barrier function using transepidermal water loss (TEWL) have proven to be difficult (67). Pinto et al. used mathematical modeling of TEWL desorption curves resulting from a plastic occlusion stress test (57). These investigators found statistically significant differences between kinetic parameters obtained from the skin of sensitive subjects compared to non-sensitive subjects in both evaporation half-life (*p* = 0.005) and dynamic water mass (*p* = 0.0001). Moreover, daily use of moisturizer (4 months in duration and expected to improve barrier function) did decrease skin sensitivity (7). In a recent study Buhé et al. found no modification of the epidermal thickness in individuals with sensitive skin compared to a non-sensitive control (4).

Decreased lipids in sensitive skin subjects have been observed for several decades. Roussaki-Schulze et al. observed that individuals with sensitive skin possess very dry skin with overall low fatness, which leads to a disturbance of the protective skin barrier function (60).

The permeability barrier in the stratum corneum depends highly on lipid composition, a more accurate predictor of skin permeability than stratum corneum thickness or cell number (68). Derangement of intercellular lipids was associated with a decline in barrier function in sensitive skin (76); specifically, decreased ceramides and sphingolipids are associated with reduced barrier integrity (14, 67). A weak barrier allows penetration of potential irritants (56), inadequately protects nerve endings, and facilitates access to antigen presenting cells, a mechanism that would support an association with atopic conditions (73). The stratum corneum barrier in sensitive skin has been demonstrated to be easily disrupted (73) with additional impairment of normal barrier recovery (77).

A recent publication by Chen et al. evaluated self-perceived sensitive skin subjects using a Lactic Acid Sting Test to evaluate the sensory response, a sodium lauryl sulfate skin irritation test to evaluate barrier function, and reactivity to DMSO to evaluate cutaneous vascular reactivity (78). These authors determined that some of the sensitive skin subjects exhibited the characteristics of high vascular reactivity without impaired barrier function.

#### Neurosensory Dysfunction

Sensitive skin is characterized on the basis of sensory responses (stinging, burning, pain, pruritus, and tingling sensations) to stimuli that normally should not provoke such sensations (5). Objective clinical signs of irritation are often absent. Of the two studies reviewed that did evaluate the relationship between neurosensory responses and objective clinical irritation, and included only subjects with demonstrated sensory sensitivity, both showed a correlation between sensory and objective signs (60, 79). A study reported in 2008 evaluated perception threshold measurement (80). Capsaicin (0.075%) and well-controlled electric currents were applied to the skin, and then sensory perception threshold was measured. Sensitive skin subjects had lower perception for c-fiber measurements than controls, suggesting the presence of a physiologically based neurological instability with modulation of c-fiber nociception as a component. In a study regarding sensitivity to facial tissue that did not exclude non-sensitive individuals, sensory effects were demonstrated to be the most reliable measure of product differences (81).

There has been little consistent correlation observed between individual response to specific irritants in testing and self-perceived sensitive skin (8). For example, sensitivity to one irritant did not predict sensitivity to others (15). Green and Shaffer, in fact, found pronounced disparity in sensitive-skin subjects with regards to irritant response to just two chemicals (82). Although one study found that those who believed their skin to be sensitive were more likely to be stingers (59%) than non-stingers (48.9%) (33).

The variety of sensory manifestations that sensitive skin patients report, combined with the scarcity of objective signs, would seem strongly to indicate the presence of neurosensory defects in sensitive skin, presumed to be related to acceleration of nerve response and therefore low sensitivity threshold (8). The pain sensations that are the hallmark of the disorder also imply possible integration dysfunctions in the central nervous system.

As reviewed by Misery et al. some studies on atopic dermatitis have shown that the density of dermal nerves is significantly higher compared to normal skin subjects; a result linked to positive sting test results in these patients (3). However, in a recent investigation by Buhé et al. cutaneous biopsies from 50 healthy women with non-sensitive or self-declared sensitive skin were systematically analyzed for a number of characteristics (4). The investigators found no modification of the epidermal thickness in sensitive skin individuals, nor did they find variations in inflammatory markers. However, there was evidence of the involvement of sensory nerve endings in individuals with sensitive skin, specifically, a decrease of intraepidermal nerve fiber density. In particular, biopsies from sensitive skin individuals exhibited a reduced peptidergic C-fiber density: the fibers involved in pain, itching and temperature perception. The authors noted a parallel with neuropathic pruritus or neuropathic pain within the context of small-fiber neuropathy and proposed that the degeneration of these fibers may promote allodynia. Altered sensations in individuals with sensitive skin might result from an insufficient protection of intraepidermal nerve fibers.

Direct connections were observed between unmyelinated nerve fibers and mast cells; stress in animal models induces substance P (SP) in unmyelinated nerve fibers that triggers mast cell degranulation with subsequent histamine release (83). Stress is commonly reported as a trigger for sensitive skin, and mast cell degranulation supported by finding that sensitive skin sufferers had higher density of mast cells and size of lymphatic microvasculature (84).

Recent research efforts are homing in on the molecular basis for sensory hyper-reactivity. Transient receptor potential vanilloid-1 (TRPV1) is a non-selective cation channel that responds to heat and low pH, and is related to nociception, neurogenic inflammation, and pruritus (71, 85). TRPV1 is expressed on fibroblasts, mast cells, and endothelial cells; activation results in pain or pruritus with a burning component (85). TRPV1 is also dramatically upregulated by inflammatory mediators (85). Ehnis-Pérez et al. obtained skin biopsies from the nasolabial fold of each of 31 subjects self-diagnosed as having sensitive skin in order to carry out analysis of TRPV1 (71). Immunohistochemistry staining for TRPV1, and TRPV1 mRNA expression was greater in subjects who tested positive in the lactic acid stinging test. The increased mRNA expression correlated with the intensity of symptoms. The authors concluded that TRPV1 expression is upregulated in subjects with sensitive skin. Sun et al. collected blood samples from individuals identified as having sensitive skin via the lactic acid stinging test (72). Samples were subjected to genetic analysis of four TRPV1 gene single nucleotide polymorphisms. The sensitive group was found to have a higher frequency of two specific TRVP1 genotypes compared to the non-sensitive group, indicating TRPV1 may have an important role in the pathogenesis of sensitive skin.

## Promising Approaches to Potential Intervention and Treatment

Following the observation that probiotic supplements can improve skin barrier function (86) and influence the pathogenesis of skin disease (87), Guéniche et al. evaluated a cream containing *Bifidobacterium longum* lysate in the treatment of sensitive (reactive) skin, measuring skin sensitivity and susceptibility by a variety of methods (77). *Ex-vivo* human skin explants were treated with both control and probiotic creams. Treated explants were significantly improved with regard to measures of inflammation such as edema, tumor necrosis factor alpha (TNF-alpha) release, and mast cell degranulation as compared to controls. Moreover, incubation of nerve cells in culture medium containing the probiotic lysate significantly inhibited capsaicin-induced calcitonin gene-related peptide (CGRP); a neuropeptide that functions in the transmission of pain.

A subsequent randomized, double-blind clinical trial studied the use of a topical cream containing *Bifidobacterium longum* extract applied to the skin of the face, arms and legs twice daily for 2 months. Reactive skin volunteers who applied the cream with bacterial extract (*n* = 33) had a significant decrease in skin sensitivity at the end of the treatment compared to reactive skin volunteers (*n* = 33) using a placebo cream. Moreover, the bacterial extract treatment led to increase skin resistance against physical and chemical aggression compared to the group of volunteers who applied the control cream (77).

Since *B. longum* lysate was shown to both decrease capsaicin-induced CGRP as well as improve barrier function, the authors speculated that the decrease in skin sensitivity observed in the clinical trial was produced by a combination of both reduced neuron reactivity and reduced accessibility of neurons (77).

Several investigators have evaluated the potential use of the bioactive trans-4-tert-butylcyclohexanol in mitigating the symptoms of sensitive skin. As reviewed in a previous section of this chapter, TRPV1 is a non-selective cation channel related to nociception, neurogenic inflammation and pruritus. Activation of TRPV1 results in pain or pruritus with a burning component. TRPV1 is expressed on fibroblasts, mast cells, and endothelial cells, and is dramatically upregulated by inflammatory mediators. Sensitive skin shows an enhanced expression of TRPV1 (71). In an investigation by Kueper et al. 30 volunteers were treated on the nasolabial fold with topical emulsions containing 31.6 ppm capsaicin with or without 0.4% trans-4-tert-butylcyclohexanol (85). In this double-blind study, 27 of the volunteers were able to sense the capsaicin-induced burning, and 26 of the 27 felt reduced burning when 0.4% trans-4-tert-butylcyclohexanol was present. The observed effect of trans-4-tert-butylcyclohexanol was highly significant (*p* < 0.0001). An additional study with 20 volunteers and a concentration of 1% trans-4-tert-butylcyclohexanol showed similar results (*p* < 0.001).

Sulzberger et al. screened several materials for the ability to both decrease release of the inflammatory mediators and inhibit TRPV1 activation (88). In *in vitro* studies these investigators used several specialized cell types to demonstrate that 4-tert-butylcyclohexanol inhibited the release of the inflammatory mediator PGE_2_ and the immune response regulator NFκB. The substance also inhibited the activation of TRPV1 in the HT1080 cell line which selectively expresses the human his-tagged TRPV1 gene. In *in vivo* studies, pretreatment of the nasolabial fold of 24 volunteers with 4-t-butylcyclohexanol significantly reduced the burning stinging response to capsaicin. In a second *in vivo* study, the forearms of 38 volunteers were shaved once daily for three consecutive days to mechanically induce barrier disruption and erythema. Test sites treated with a solution containing 4-tert-butylcyclohexanol showed significantly reduced redness compared to control sites.

Phenoxyethanol is a common preservative in personal care products. This substance can induce skin discomfort in some individuals. Li et al. screened 243 Chinese female subjects and found that 60 experienced burning and itching sensations in response to 1% phenoxyethanol (89). In a double-blinded, randomized study, a formulation containing phenoxyethanol was applied to the nasolabial fold on one side of the face, and a formulation containing both phenoxyethanol and trans-tert-butyl cyclohexanol was applied to the other side. Results confirmed that phenoxyethanol induced a skin burning and itching sensations in these subjects. The uncomfortable skin sensations were significantly inhibited by trans-tert-butyl cyclohexanol.

## Conclusion

Sensitive skin is defined by the occurrence of unpleasant sensations in response to stimuli that normally should not provoke such sensations, and which cannot be explained by lesions attributable to any skin disease (5). This condition continues to be somewhat of a medical enigma with no correlation between sensory symptoms and subjective signs, and no reliable diagnostic test. Although it is clear that specific individuals clearly have heightened sensitivity to different kinds of sensory and physical irritants, observed reactions are not predictive of generalized sensitivity and the relationship between observed sensitivities is unclear (41, 90).

The prevalence of sensitive skin varies in different geographies and cultures, but it is generally agreed that this condition effects a substantial portion of the population. A variety of host–related factors (i.e., skin type, gender and hormonal factors), and external factors (i.e., climate, exposures to products and chemicals, and cultural influences) contribute to self-perceived sensitive skin. Investigations have pointed to two general physiological causes of sensitive skin. It is clear that sensitive skin exhibits alterations in barrier function with derangement of intercellular lipids and a decrease in ceramides and sphingolipids resulting in reduced barrier integrity. Neurosensory changes are also evident with a heightened sensory input and upregulation of the non-selective cation channel TRPV1.

Current needs are to continue to pursue reliably predictive methodologies to diagnose sensitive skin, as well as methods capable of detecting very subtle skin benefits or the potential for slight adverse effects. In addition, global epidemiological data must continue to be bolstered by studies that build on what is known about the physiological components of sensitive skin.

## Author Contributions

MAF primary author on writing and review of the manuscript.

### Conflict of Interest Statement

MAF is an employee of the Procter and Gamble Company.
